# DLGRAFE-Net: A double loss guided residual attention and feature enhancement network for polyp segmentation

**DOI:** 10.1371/journal.pone.0308237

**Published:** 2024-09-12

**Authors:** Jianuo Liu, Juncheng Mu, Haoran Sun, Chenxu Dai, Zhanlin Ji, Ivan Ganchev

**Affiliations:** 1 College of Mathematics and Computer Science, Zhejiang A&F University, Hangzhou, China; 2 Hebei Key Laboratory of Industrial Intelligent Perception, North China University of Science and Technology, Tangshan, China; 3 Telecommunications Research Centre (TRC), University of Limerick, Limerick, Ireland; 4 Department of Computer Systems, University of Plovdiv “Paisii Hilendarski”, Plovdiv, Bulgaria; 5 Institute of Mathematics and Informatics—Bulgarian Academy of Sciences, Sofia, Bulgaria; Shijiazhuang Tiedao University, CHINA

## Abstract

Colon polyps represent a common gastrointestinal form. In order to effectively treat and prevent complications arising from colon polyps, colon polypectomy has become a commonly used therapeutic approach. Accurately segmenting polyps from colonoscopy images can provide valuable information for early diagnosis and treatment. Due to challenges posed by illumination and contrast variations, noise and artifacts, as well as variations in polyp size and blurred boundaries in polyp images, the robustness of segmentation algorithms is a significant concern. To address these issues, this paper proposes a Double Loss Guided Residual Attention and Feature Enhancement Network (DLGRAFE-Net) for polyp segmentation. Firstly, a newly designed Semantic and Spatial Information Aggregation (SSIA) module is used to extract and fuse edge information from low-level feature graphs and semantic information from high-level feature graphs, generating local loss-guided training for the segmentation network. Secondly, newly designed Deep Supervision Feature Fusion (DSFF) modules are utilized to fuse local loss feature graphs with multi-level features from the encoder, addressing the negative impact of background imbalance caused by varying polyp sizes. Finally, Efficient Feature Extraction (EFE) decoding modules are used to extract spatial information at different scales, establishing longer-distance spatial channel dependencies to enhance the overall network performance. Extensive experiments conducted on the CVC-ClinicDB and Kvasir-SEG datasets demonstrate that the proposed network outperforms all mainstream networks and state-of-the-art networks, exhibiting superior performance and stronger generalization capabilities.

## 1. Introduction

Deep learning (DL) has greatly improved the performance of automatic image segmentation in medical diagnosis. As a new research direction in the field of artificial intelligence, deep learning has been widely applied and researched in the field of medical image segmentation [1–4]. With the continuous advancement of AI, a series of new methods are emerging in the healthcare sector to improve diagnostic accuracy and efficiency. Currently, cancer is a prominent area of research due to its complexity characterized by multiple genetic and epigenetic variations, and it ranks as the second leading cause of death globally [5,6]. By implementing appropriate prevention, early detection, and treatment strategies, approximately 3.7 million lives could be saved annually [7,8]. It’s estimated that over one-third of death cases due to cancer can be prevented with timely interventions [9].

Colorectal cancer is a malignant tumor that originates in the colon or rectum, typically forming within the walls of the intestine [10,11]. This type of cancer tends to progress slowly, and initially, there may be no apparent symptoms. However, the chances of cure are higher when it is detected early through endoscopic examination and if promptly treated [12,13]. The endoscopy may sometimes cause the doctor to miss some potentially cancerous polyps due to the similar color of the polyps and the background. In order to solve this problem, the use of computer-based deep learning to assist doctors in diagnosis has become particularly important.

With the continuous expansion and development of deep learning applications, an increasing number of deep learning-based segmentation methods have been proposed recently [14–17]. In cases where a doctor may have overlooked polyp regions, these segmentation methods can perform additional scans to guide the reanalysis of pathological information at that location. This, in turn, enhances a comprehensive assessment of the patient, facilitating the more effective detection and management of potential precancerous lesions. While these methods have made progress, the segmentation accuracy is compromised because polyps exhibit low contrast and similar colors to the surrounding environment, making it challenging to effectively determine the boundaries of polyp contours [4,18,19].

Inspired by the architectures of fully convolutional networks and ResNet, this paper introduces a Double Loss Guided Residual Attention and Feature Enhancement Network (DLGRAFE-Net) for polyp segmentation tasks [20,21]. The proposed network utilizes ResNet34 in the encoder to extract features, whereas the decoder utilizes the newly designed Efficient Feature Extraction (EFE) modules. Additionally, the newly designed Semantic and Spatial Information Aggregation (SSIA) module and Deep Supervision Feature Fusion (DSFF) modules are used to obtain local loss for the network and perform feature fusion.

The main contributions of this paper can be summarized as follows:

1. A Double Loss Guides Residual Attention and Feature Enhancement Network (DLGRAFE-Net) is proposed, which utilizes a pre-trained ResNet34 in the encoder and a novel EFE-based decoder;
2. A newly designed SSIA module is proposed, leveraging richer spatial information in low-level feature graphs and more abundant semantic information in high-level feature graphs, employing standard square convolution to extract features from the low-level and high-level feature graphs of the encoding structure, and generating local loss to guide the network training;
3. Newly designed DSFF modules are introduced for addressing the negative impact caused by the imbalance in the background due to varying polyp sizes, which performs feature fusion by combining the local loss feature graph with multi-level features from the encoder and uses the local loss feature graph to guide the network in obtaining the most extensive features from different encoding layers;
4. Newly designed EFE decoding modules are proposed to address the issue of losing important information in the feature graphs of the decoding layers, which allows the network to selectively weigh the importance of each channel, generate more crucial information in the output, and facilitate more accurate polyp segmentation.

## 2. Materials and methods

### 2.1 CNN-based polyp segmentation

ResNet (Residual Network) is a specialized architecture within Convolutional Neural Networks (CNNs) [20]. In contrast to typical CNN structures, ResNet introduces residual units with identity mappings [22]. In conventional deep neural networks, as the number of layers increases, issues such as vanishing or exploding gradients can arise, making it challenging for the network to converge. To address this problem, ResNet introduces the concept of residual units [4]. Residual units allow the network to directly learn the shallow-layer input and then focus on learning the differences in the deeper layers, transforming the learning problem into one of learning residuals, [23]. This simplifies the network’s learning process. PraNet [24], proposed by Fan et al., is one of the most classic network structures in the field of polyp segmentation. To address the issue of unclear boundaries, these authors first utilize a Parallel Partial Decoder (PPD) to aggregate features from higher layers. Then, based on the combined features, they generate a global map as the initial guidance region for the subsequent components. Additionally, a Reverse Attention (RA) module is employed to explore boundary cues, establishing relationships between regions and boundary clues. Liu et al. proposed a thick and fine segmentation framework for polyp segmentation, based on depth and classification features [25]. In order to improve the accuracy of polyp segmentation, the prediction graph of complex samples was used as prior information to guide the evolution of active contour models.

ConvSegNet [26], proposed by Ige et al., introduces a novel Context Feature Refinement (CFR) module. This module extracts context information from the incoming feature map using parallel convolution layers with different kernel sizes. This enables the network to effectively identify and segment both small details and larger, more complex, structures in the input images. Recently, there has been a growing trend in proposing networks based on the Transformer architecture for medical image segmentation, following its introduction by Vaswani et al. in 2017 [27]. Based on Transformer, Yang et al. proposed TranSEFusionNet to address the limitations of U-Net in medical image segmentation [28] and to reduce losing the information during the polyp image feature fusion.

Liu et al. introduced ECTransNet [29] in 2023, incorporating an Edge Complementary module and utilizing the Transformer structure. This module effectively fuses differences between features with various resolutions, allowing the network to exchange features across different levels and significantly enhancing edge details in polyp segmentation. Furthermore, the authors employ a feature aggregation decoder, adaptively merging high-level and low-level features using residual blocks. This strategy preserves target spatial information in high-level features while restoring local edges in low-level features, ultimately improving segmentation accuracy [30]. However, when analyzing polyp images by transforming them into word vectors, Transformer faces a challenge—it may lose the original image’s positional information. In tasks such as polyp segmentation, positional information is crucial for accurate analysis. Compared to fully convolution-al networks, the Transformer architecture may not perform optimally in capturing local information in polyp images [31].

### 2.2 Datasets

For the polyp image segmentation task, each pixel in the training images is labeled as either a polyp or non-polyp. The evaluation of DLGRAFE-Net was performed based on experiments conducted on the Kvasir-SEG [32] dataset and the CVC-ClinicDB [33] dataset. The Kvasir-SEG dataset consists of 1000 polyp images along with their corresponding annotated maps. These images were annotated by expert endoscopists at Oslo University Hospital. The CVC-ClinicDB dataset comprises 612 polyp images. The training set, used in the experiments, was composed of 900 images from Kvasir-SEG and 550 images from CVC-ClinicDB, with a total of 1450 images, which were randomly selected. There were two test sets, composed of the remaining 100 images from Kvasir-SEG and 62 images from CVC-ClinicDB. There were no duplicate images in the training set and test set.

### 2.3 Proposed network

#### 2.3.1 Overall architecture

**Fig 1:**
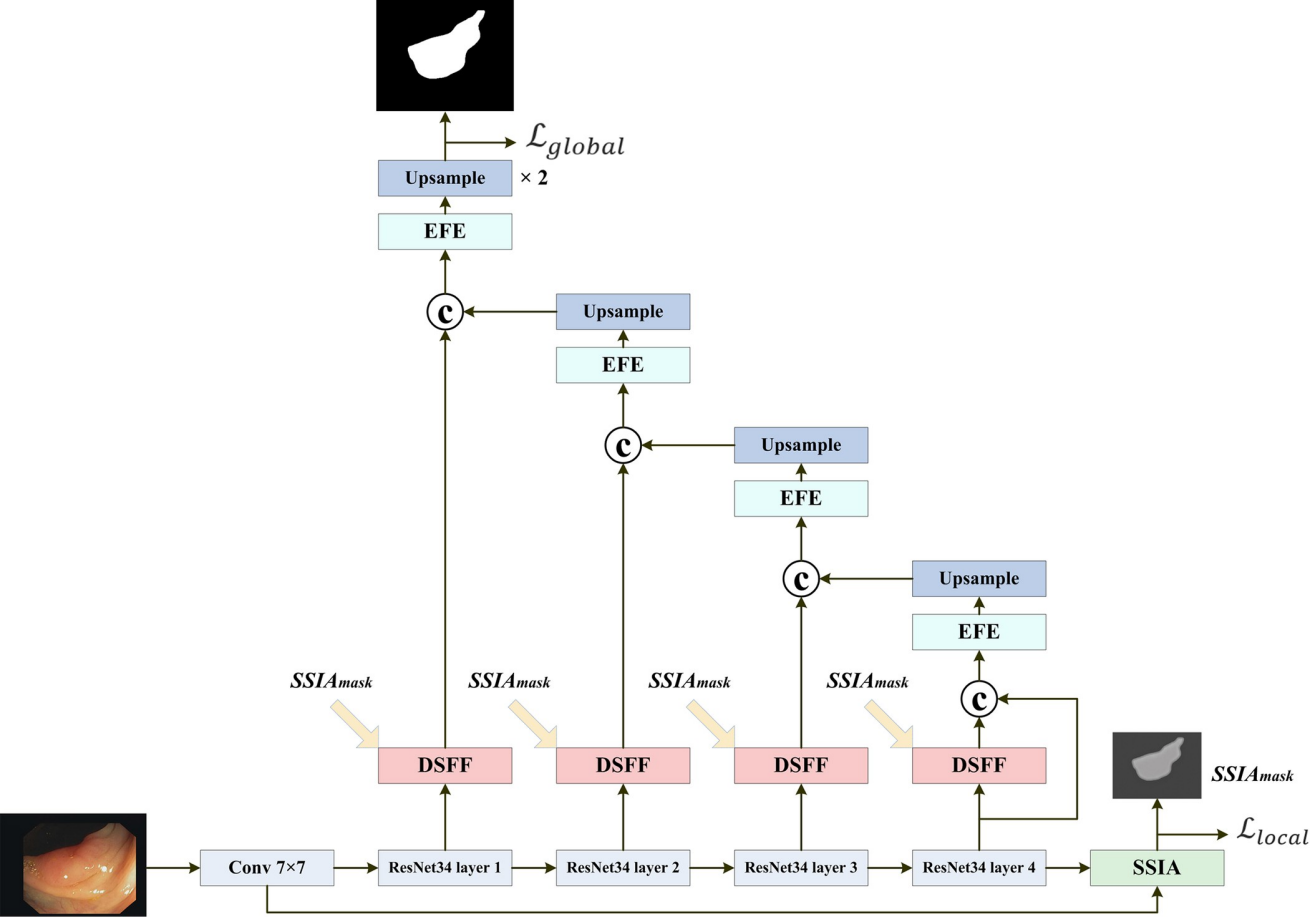
The architecture of the proposed DLGRAFE-Net network.

Based on a fully CNN architecture, the proposed DLGRAFE-Net network includes three new types of modules, as illustrated in Fig 1.

The polyp images are encoded using a 7×7 convolution and the ResNet34’s BasicBlock residual module (in a four-layer structure) due to its moderate depth and strong feature extraction capabilities, as well as the advantages of residuals connections used, ensuring that gradients can be transmitted efficiently. A 7×7 convolution is used in order to capture large local features in the input images. Compared to smaller convolution kernels, 7×7 convolution kernels are able to cover a larger area in the images in a single operation, which helps capture large-scale features. This is especially important in medical images because polyps vary widely in size and shape. A filter set to [32, 64, 128, 256, 512] is used, according to the hardware configuration. A newly designed SSIA module is utilized to gather richer spatial and semantic feature information, which is fused to extract local features, reconstruct mask prediction results, learn module weight parameters, and update gradients through a *local* loss function defined in [18]. Newly designed DSFF modules perform feature fusion between local feature graphs and multi-level features from the encoder, focusing the network training on strongly relevant regions. These modules transfer the weights of local feature graphs to the encoder, supervise the encoder network training, and ultimately utilize a novel EFE-based decoder, which simultaneously considers global relationships and spatial details, to reconstruct higher-resolution segmentation results [34].

The encoder module performs down-sampling on input images and extracts essential features. The encoder path is composed of a pre-trained ResNet34. Each residual block consists of two 3×3 convolutions with a stride of 2. Specifically, the four ResNet34 layers are made up of 3, 4, 6, and 3 residual blocks, respectively. The output *E*_*j*_ of the *j*-th residual block is produced as follows:

$$E_{j}=Conv3\times 3(Conv3\times 3(X))+Conv3\times 3(X)$$ (1)

where *Conv*3×3denotes a 3×3 convolution with a stride of 2 and *X* denotes the current input to the convolutional layer.

The output *SSIA*_*mask*_ of the SSIA module is used for updating gradients, through a *local* loss function defined in (19), in order to concentrate local feature graph information on strongly relevant regions. The SSIA output is formed as follows:

$${SSIA}_{mask}=\rho [Conv7\times 7(X),E_{16}]$$ (2)

where *Conv*7×7 denotes a 7×7 two-dimensional (2D) convolution with a step length of 2, *ρ* denotes the feature fusion operation, and *E*_16_ denotes the output of the 16-th residual block.

Each DSFF module fuses *SSIA*_*mask*_ with the different scale features of the encoder, enabling the network to capture remote relationships while focusing on the training of areas of strong interest [34]. Due to the guidance of *SSIA*_*mask*_, the encoder training can alleviate the negative effects of background imbalance caused by different polyp sizes. The output *DSFF*_*j*_ (*j* = 3, 7, 13, 16) of a DSFF module after feature fusion between the *j*-th residuals and *SSIA*_*mask*_ is produced as follows:

$${DSFF}_{j}=\rho [{SSIA}_{mask},\;E_{j}]$$ (3)

where *E*_*j*_ denotes the output of the *j*-th residuals.

Unlike the encoder path, the decoder path is composed of a series of EFE modules for feature extraction. The output of each DSFF module is spliced with a corresponding up-sampled EFE module to further refine the output features of the module. The up-sampling unit with scale of 2 is used to up-sample the feature graphs received from the lower network layer. The output *D* of the decoding phase is produced as follows:

up=Upsample(X,2) (4)

$$D=EFE\{concat[up,{DSFF}_{j}]\}$$ (5)

where *up* denotes the up-sampled output, *EFE* denotes the decoder, and *concat* denotes the operation of joining the features of the same size together.

#### 2.3.2 SSIA module

**Fig 2:**
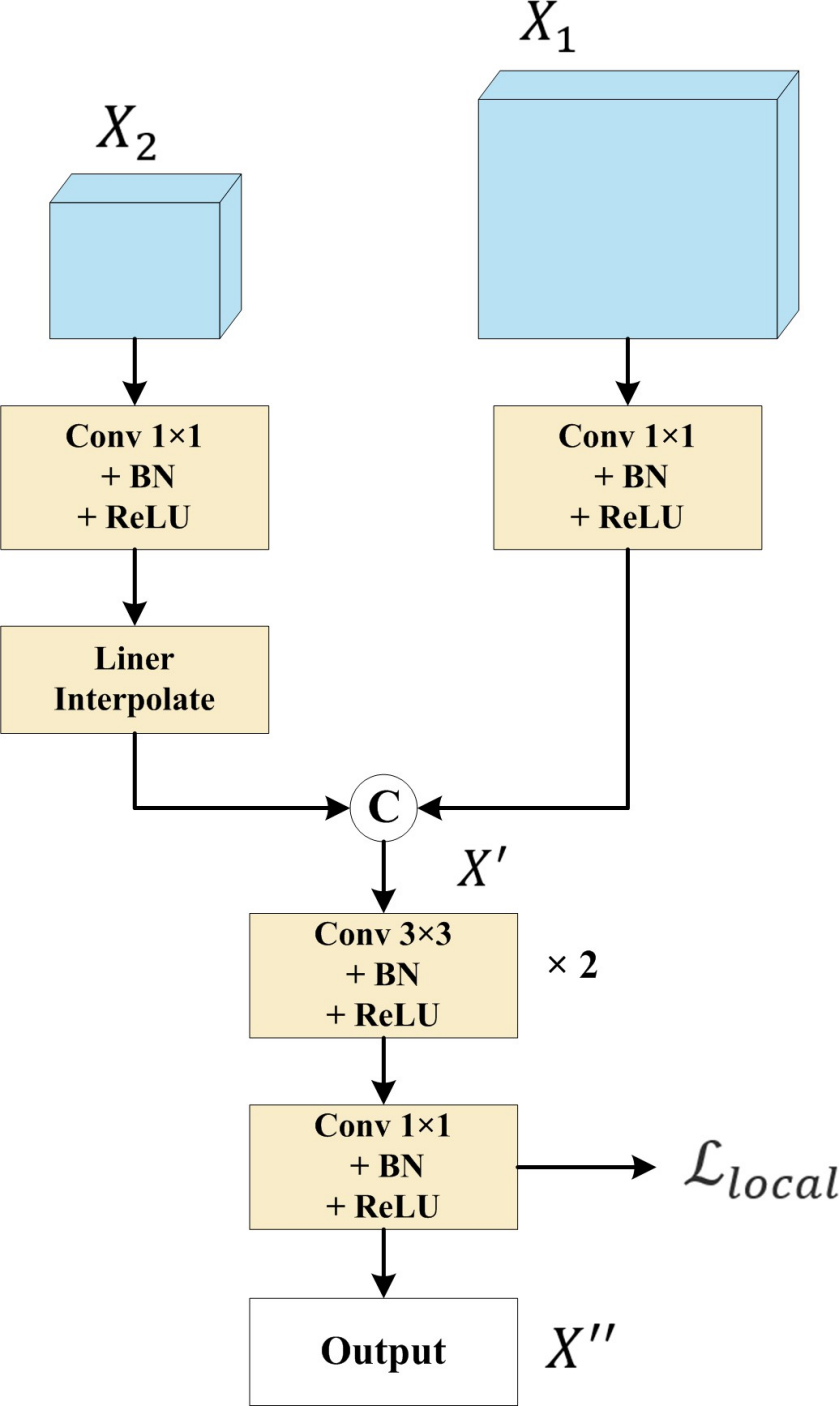
The newly designed SSIA module structure, utilized by the proposed DLGRAFE-Net network.

To accurately extract polyps from colonoscopy images, a newly designed SSIA module, depicted in Fig 2, is utilized, which takes advantage of the richer spatial information in low-level feature graphs and the more abundant semantic information in high-level feature graphs obtained from deep learning.

The SSIA module utilizes a convolutional structure to fuse low-level and high-level features, reconstructing mask prediction results. It combines deep coarse global features with shallow detailed global features to generate local feature graphs, reducing the aliasing effect caused by down-sampling. Simultaneously, the module learns weight parameters and updates gradients through a *local* loss function defined in (18). The SSIA module performs the following computations:

$$X\prime =concat(Conv1\times 1(X_{1}),\tau [Conv1\times 1(X_{2})])$$ (6)

$$X^{\prime \prime }=Conv\;1\times 1\{Conv3\times 3[Conv3\times 3(X\prime )]\}$$ (7)

where *X*_1_ and *X*_2_ denote the two input feature graphs, respectively, *τ* denotes an interpolation operation, *concat* denotes a channel concatenation operation, *X*′ denotes the intermediate output feature graph, *X*′′ denotes the final output feature graph, *Conv*3×3 denotes a 2D convolution with a convolution kernel of 3, and *Conv*1×1 denotes a 2D convolution with a convolution kernel of 1.

#### 2.3.3 DSFF modules

**Fig 3:**
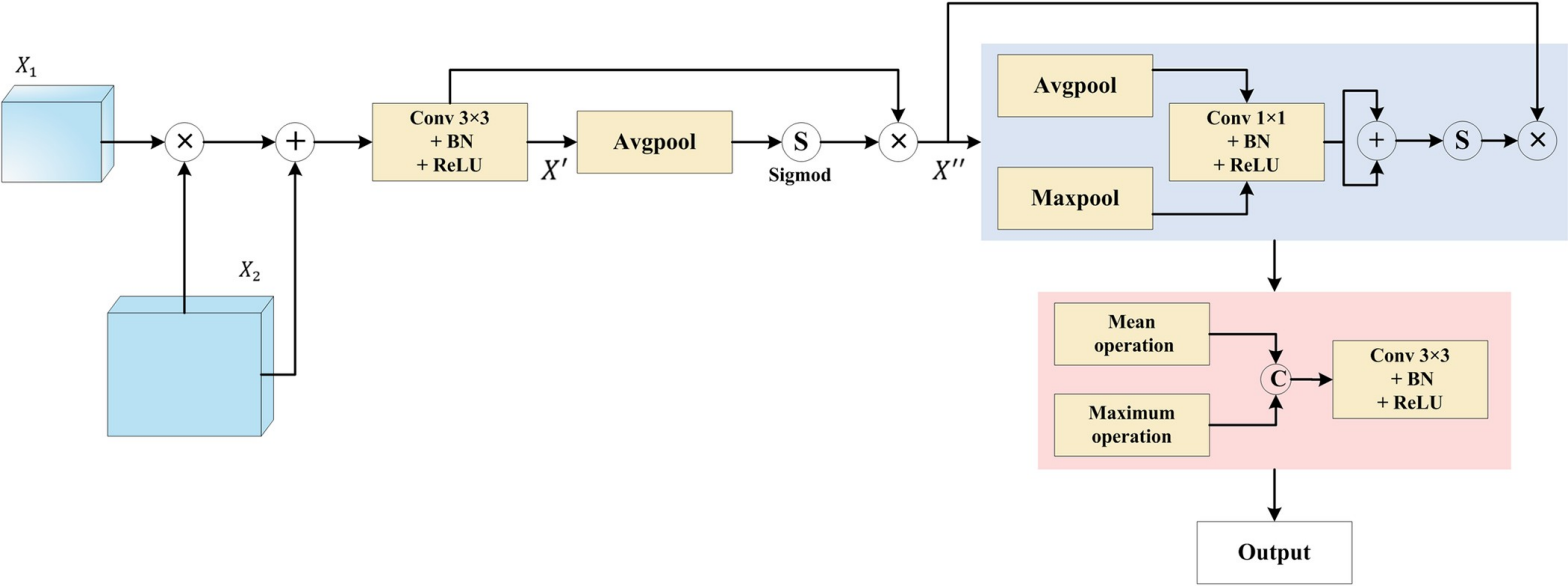
The newly designed DSFF module structure, utilized by the proposed DLGRAFE-Net network.

In the convolutional feature extractor, newly designed DSFF modules are used to increase the receptive field of convolutional features. Guided by the local feature graph, each DSFF module extracts strongly-relevant region features from multiple scales of the encoder, reduces weights in irrelevant regions, and alleviates the negative impact caused by the imbalance in polyp background. The use of a 1×1 2D convolution imparts non-linearity to the feature graphs, broadening the network’s capabilities. This is why a "deep" network is often preferred over a "wide" network. Finally, the two processed feature graphs are fused by concatenation and refined further using two 3×3 convolutions. The DSFF structure is illustrated in Fig 3. Each DSFF module performs the following computations:

$${X^{\prime }=Conv3\times 3[X}_{1}⨀\tau (X_{2})+X_{2}]$$ (8)

$$X^{\prime \prime }=\sigma [Avgpool(X^{\prime })]⨀X^{\prime }$$ (9)

where *X*_1_ and *X*_2_ denote the two input feature graphs respectively, *σ* denotes the sigmoid operation, ⨀ denotes an element-wise multiplication, *X*′ denotes the intermediate output feature graph, *X*′′ denotes the final output feature graph, *Avgpool* indicates the average pooling operation.

#### 2.3.4 EFE modules

**Fig 4:**
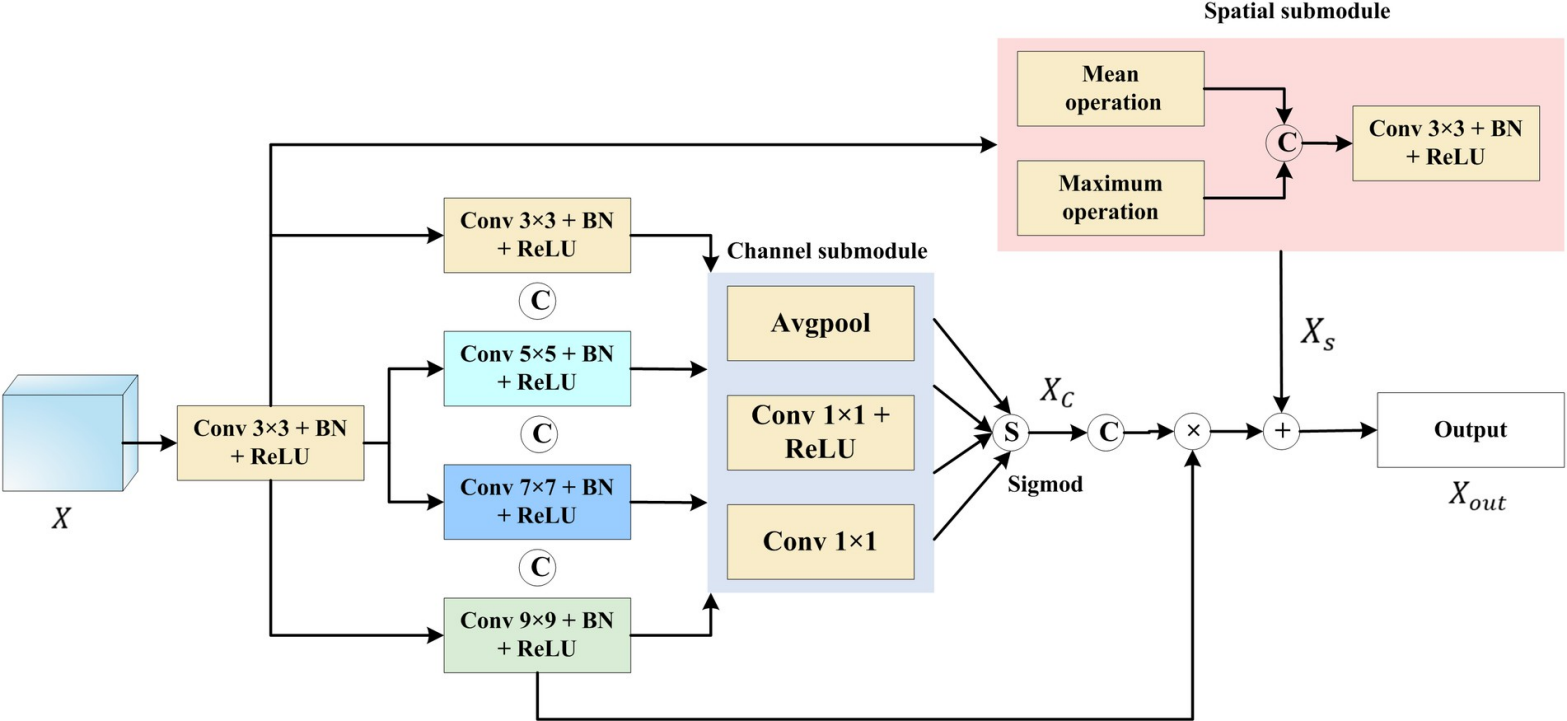
The newly designed EFE module structure, utilized by the proposed DLGRAFE-Net network.

The main function of the EFE decoding modules is to reconstruct higher-resolution segmentation results based on the spatial relationships extracted by the encoder and the semantic spatial features obtained from the convolutional branch. It achieves this through up-sampling, capturing different semantic features using a multi-scale channel and spatial attention mechanism. This mechanism is highly effective in capturing local features, allowing the network to disregard obvious global information during the decoding process, thereby emphasizing local complexity and highlighting the boundaries of the segmentation targets. The EFE structure is illustrated in Fig 4. Each EFE module performs the following computations:

$$X_{i(i=1,2,3,4)}=Conv\;N\times N(X)\;(N=3,5,7,9)$$ (10)

$$X_{C(C=1,2,3,4)}=Channel(X_{i(i=1,2,3,4)})$$ (11)

$$X_{s}=Spatial(X)$$ (12)

$$X_{out}=concat(X_{i(i=1,2,3,4)})⨀[concat(X_{C(C=1,2,3,4)})+X_{s}]$$ (13)

where *X* denotes the input feature graph, *X*_*out*_ denotes the output feature graph, *Conv N*×*N* denotes a 2D convolution operation with a convolution kernel of *N*, *Channel* denotes the operation performed by the channel submodule, and *Spatial* denotes the operation performed by the spatial submodule. The spatial submodule converts various deformation data in space and automatically captures important regional features, whereas the channel submodule forms the importance of each channel through feature learning, and finally assigns different weights to each channel.

### 2.4 Loss functions and experimental setup

The proposed DLGRAFE-Net network utilizes a combined BCE-Dice loss [35] in a *global* loss function, in order to provide finer grained gradient information for the whole network training, along with improving its stability and sensitivity. The BCE-Dice loss combines the Binary Cross Entropy (BCE) loss [9] with the Dice loss [36], which are commonly used in binary segmentation tasks. The BCE loss is a loss function used to measure the disparity between a network’s output and the actual labels in binary classification problems. For each sample, the BCE loss computes the cross-entropy loss between the probability distribution predicted by the network and the actual labels, and then averages the losses across all samples. The Dice loss performs well in scenarios with severe imbalance of positive and negative samples, emphasizing foreground region exploration during the network training process. Utilizing these two loss functions in the global network can effectively assist in learning accurate segmentation.

The BCE loss is defined in [9], as follows:

$$BCE(p,q)=-\frac{1}{N}\sum_{i=1}^{N}\left(q_{i}\times log(p_{i})+(1-q_{i})\times log(1-p_{i})\right)$$ (14)

where *N* denotes the number of pixels, *q*_*i*_ denotes the actual label of the *i*-th pixel (0 or 1), and *p*_*i*_ denotes the predicted probability that the *i*-th pixel belongs to class 1.

The Dice loss is calculated, as per [37], as follows:

$$Dice(p,q)=\frac{2\sum_{i=1}^{N}{(p}_{i}\times q_{i})}{\sum_{i=1}^{N}p_{i}^{2}+\sum_{i=1}^{N}q_{i}^{2}}$$ (15)

where *q*_*i*_ denotes the target label of the *i*-th pixel, i.e., the binarized true label.

The combined BCE-Dice loss is calculated as follows:

BCE_Dice(p,q)=α×BCE(p,q)+(1−α)×Dice(p,q)) (16)

where *α* denotes the weight factor with a value set to 0.5, based on experiments confirming that the network training reaches top performance when *α* = 0.5.

The *global* loss function (c.f., Fig 1), used for training the proposed network, is defined as follows:

$$L_{global}=BCE\_Dice(p,q)+Dice(p,q)$$ (17)

In addition, a *local* loss function is used in the local feature graph passing through the SSIA module of the proposed network (c.f., Fig 1), as it can effectively emphasize the overlap area between the prediction results and the real labels in the coding stage, handle the category imbalance between local features, and assist the global loss function to optimize segmentation performance. The *local* loss function is defined as follows:

$$L_{local}=Dice(p,q)$$ (18)

The utilized double loss, c.f. (17) and (18), can guide the proposed network to perform better in complex image segmentation tasks.

In the proposed network, the newly designed SSIA module is employed to integrate low-level semantic features extracted by the first convolutional layer (*Conv*7×7) with deep-level semantic features obtained from the ResNet34-based encoder. Subsequently, the loss defined in (18) is utilized as a *local* loss function, with a specific focus on addressing sample imbalance issues during the encoding stage. This approach ensures the comprehensive extraction of meaningful target feature information, guiding the decoding process effectively.

The Adam optimizer [38] is used, which adaptively adjusts the learning rate based on the historical gradient information of different parameters by calculating the first and second moment estimates of the gradient, which allows it to converge quickly and avoid falling into local minima during the network training.

The hardware configuration used in the conducted experiments utilized an Intel Core i5-12490 processor with a clock speed of 3.0 GHz, and a single NVIDIA RTX3060 graphics card with 12 GB memory. The hyperparameters for network training were set as follows: Batch_Size = 4, Epochs = 200 (validation was performed on each epoch, and the network was trained using the Adam optimizer), Initial_Learning_Rate = 1×10^−4^, momentum = 0.9, Minimum_Learning_Rate = 1×10^−5^. The network structure was implemented using PyTorch.

### 2.5 Evaluation metrics

In order to objectively evaluate the network performance, training was conducted on the same dataset while keeping certain parameters constant. Common metrics such as Dice Similarity Coefficient (*DSC*), *precision*, *recall*, and Intersection over Union (*IoU*) were used to evaluate the results. These metrics are defined as follows:

$$\mathrm{DSC}=\frac{2TP}{2\times TP+FP+FN}$$ (19)

$$Precision=\frac{TP}{TP+FP}$$ (20)

$$Recall=\frac{TP}{TP+FN}$$ (21)

$$IoU=\frac{TP}{FN+TP+FP}$$ (22)

where TP denotes the true positive counts, FP denotes the false positive counts, and FN denotes the false negative counts. These selected metrics provide a comprehensive evaluation of the segmentation results, enabling a fair comparison of different networks performed on the same dataset.

## 3. Experiments and results

### 3.1 Performance comparison with classic segmentation networks

**Fig 5:**
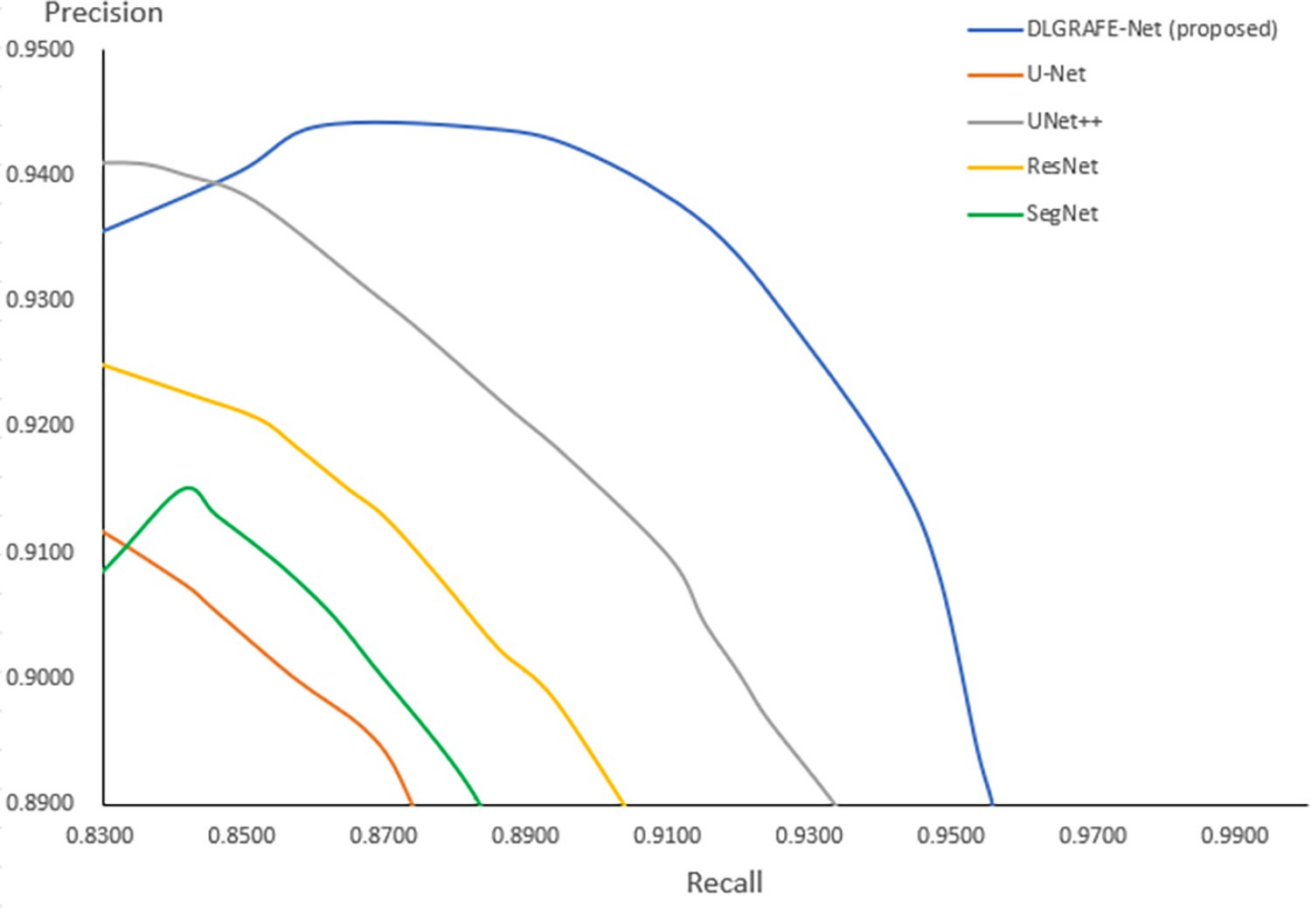
The *precision*-*recall* curve of the network training process.

In this set of experiments, the segmentation performance of the proposed DLGRAFE-Net network was compared with that of classic segmentation networks, including U-Net [21], UNet++ [39], ResNet [20], and SegNet [40]. The obtained results, shown in Tables 1 and 2, demonstrate that the proposed network outperforms all other networks according to all evaluation metrics. More specifically, on the CVC-ClinicDB dataset, the first runner-up is outperformed by 6.80, 3.83, 7.43, and 10.39 percentage points, based on *DSC*, *precision*, *recall*, and *IoU*, respectively. And on the Kvasir-SEG dataset, the first runner-up is outperformed by 3.42, 2.49, 3.51, and 4.99 percentage points, based on *DSC*, *precision*, *recall*, and *IoU*, respectively. The *precision*-recall curves, shown in Fig 5, further illustrate the superiority of the proposed DLGRAFE-Net network over the classical segmentation networks.

**Table 1:** Segmentation performance comparison results, obtained on CVC-ClinicDB dataset.

Network	*DSC* (%)	*Precision* (%)	*Recall* (%)	*IoU* (%)
U-Net	83.64	89.52	82.44	74.99
UNet++	86.13	90.41	85.21	77.79
ResNet	87.58	89.87	87.67	79.57
SegNet	84.38	88.27	85.18	75.39
DLGRAFE-Net (*proposed*)	**94.38**	**94.24**	**95.10**	**89.96**

**Table 2:** Segmentation performance comparison results, obtained on Kvasir-SEG dataset.

Network	*DSC* (%)	*Precision* (%)	*Recall* (%)	*IoU* (%)
U-Net	82.86	90.25	80.55	73.62
UNet++	84.42	87.48	85.67	76.11
ResNet	88.24	92.95	85.84	80.65
SegNet	85.75	89.53	85.11	77.41
DLGRAFE-Net (*proposed*)	**91.66**	**95.44**	**89.35**	**85.64**

In order to further verify the generalization ability and robustness of the proposed network, we have tested it, along with other classical networks, on a previously unseen dataset, CVC-300 (containing 60 polyp images), which is different from the training set used. The obtained results, shown in Table 3, demonstrate that the proposed network outperforms all classical networks, according to all evaluation metrics.

**Table 3:** Segmentation performance comparison results, obtained on CVC-300 dataset.

Network	*DSC* (%)	*Precision* (%)	*Recall* (%)	*IoU* (%)
U-Net	74.11	76.55	77.87	67.34
UNet++	80.05	78.05	90.69	72.13
ResNet	82.69	77.47	93.07	75.07
SegNet	80.98	81.94	84.57	74.22
DLGRAFE-Net (*proposed*)	**88.97**	**85.48**	**95.00**	**81.81**

**Fig 6:**
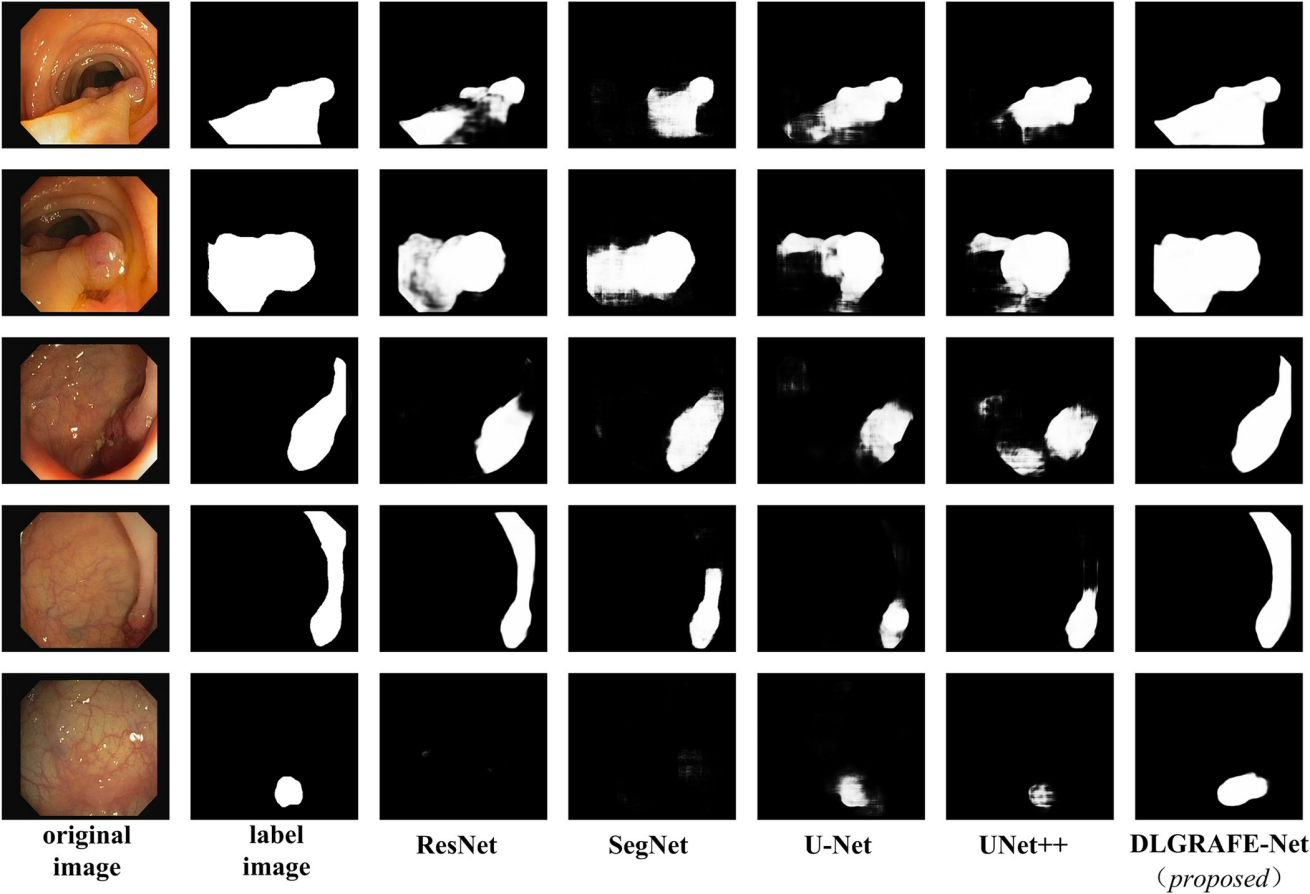
Sample visualizations of polyp segmentation performance of different networks on CVC-ClinicDB dataset.

**Fig 7:**
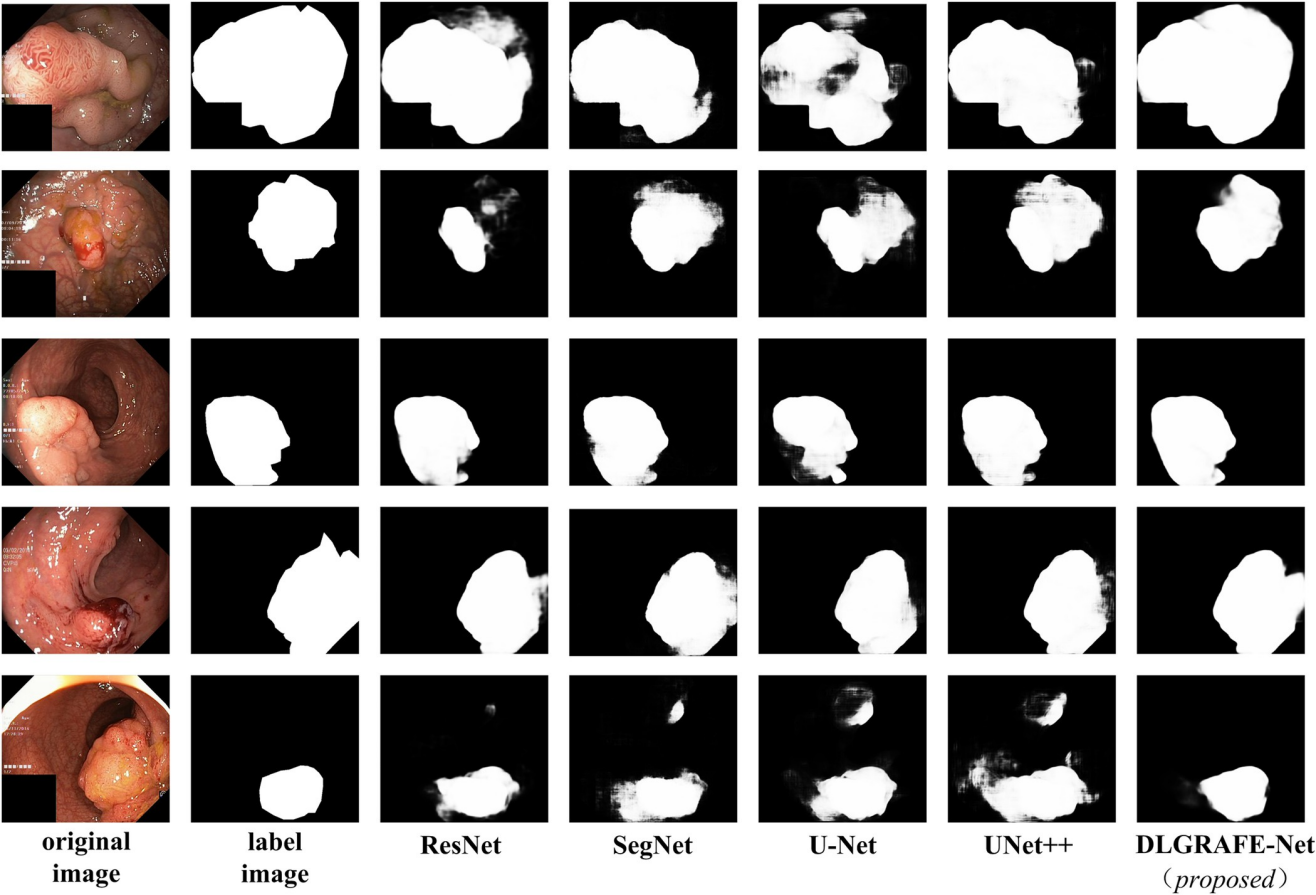
Sample visualizations of polyp segmentation performance of different networks on Kvasir-SEG dataset.

Figs 6 and 7 display predicted images output by different segmentation networks participating in this set of experiments. From these figures, it can be observed that the proposed DLGRAFE-Net network demonstrates more accurate segmentation of polyps. In comparison to other networks, DLGRAFE-Net excels in extracting features related to polyps and effectively mitigates the influence of similar background information around the polyps.

Additionally, it could be observed that when polyps are relatively small and their color is similar to the background color, the classical networks, ResNet and SegNet, lack global context information and the interaction of multi-scale features. Therefore, these networks may not accurately detect the location of polyps or even recognize the existence of polyps. In most of compared networks, boundary ambiguity and incomplete polyp segmentation appear in large-polyp segmentation. In the proposed network, a multi-scale spatial channel mechanism, provided by the EFE-based decoder, is used to capture global context information, and a cross-scale feature interaction strategy of DSFF modules is used to integrate multi-stage features well, which allows the network to achieve good results in global and local feature extraction and recovery. According to the visual renderings, the proposed DLGRAFE-Net network achieves good segmentation results when the shape of the lesion area is irregular, the boundary is blurred, and the color is similar. Overall, DLGRAFE-Net proves to be more efficient in extracting detailed features of polyps, thereby achieving much better segmentation performance than the other networks compared.

### 3.2 Ablation studies

In order to assess the effectiveness of different modules, newly designed for DLGRAFE-Net, multiple ablation study experiments were conducted on the Kvasir-SEG and CVC-ClinicDB datasets. The encoder of the proposed network is based on ResNet, whereas its decoder is based on U-Net. In these experiments, the newly designed EFE, SSIA, and DSFF modules were sequentially added in each step, as to compare it to the previous step. The obtained results are presented in Tables 4 and 5.

**Table 4:** Ablation study results, obtained on CVC-ClinicDB dataset.

Network components	*DSC* (%)	*Precision* (%)	*Recall* (%)	*IoU* (%)
Baseline	87.58	89.87	87.67	79.57
Baseline + EFE	89.20	89.86	90.51	81.78
Baseline + EFE + SSIA (without ℒ_*local*_)	90.28	92.87	90.55	84.39
Baseline + EFE + SSIA (with ℒ_*local*_)	91.97	93.18	91.71	85.77
Baseline + EFE + SSIA (without ℒ_*local*_) + DSFF	93.06	93.53	93.70	87.97
Baseline + EFE + SSIA (with ℒ_*local*_) + DSFF	**94.38**	**94.24**	**95.10**	**89.96**

**Table 5:** Ablation study results, obtained on Kvasir-SEG dataset.

Network components	*DSC* (%)	*Precision* (%)	*Recall* (%)	*IoU* (%)
Baseline	88.24	92.95	85.84	80.65
Baseline + EFE	88.83	89.50	**90.36**	82.04
Baseline + EFE + SSIA (without ℒ_*local*_)	89.78	94.38	87.86	83.56
Baseline + EFE + SSIA (with ℒ_*local*_)	90.36	94.55	88.53	83.97
Baseline + EFE + SSIA (without ℒ_*local*_) + DSFF	91.18	94.74	89.69	85.20
Baseline + EFE + SSIA (with ℒ_*local*_) + DSFF	**91.66**	**95.44**	89.35	**85.64**

As shown in Tables 4 and 5, adding the EFE modules to the baseline, in the first step, resulted in respective increase of all evaluation metrics, except for *precision*, on both datasets. This indicates that the EFE-based decoder effectively utilizes multi-scale channel and spatial attention mechanisms to reconstruct different semantic features, preserving multi-scale information. It also better distinguishes features in different directions in the images and more effectively captures information in specific directions. Continuing with the addition of the SSIA module (without applying the *local* loss function) in the second step, led to further increase of all evaluation metrics, except for *recall* on the Kvasir-SEG dataset. This is due to the fact that the boundaries of polyp regions in Kvasir-SEG are fuzzier, compared to those in CVC-ClinicDB, and the leakage rate of pixels is increased, resulting in an increase in the number of false negatives (FN), which led to reducing the *recall*. However, this relatively small drop in *recall* can be sacrificed in order to greatly improve the values of all other metrics, which also suggests that the feature graphs, locally generated by the SSIA module, allow to effectively reduce the aliasing effects caused by down-sampling. The *local* loss function defined in (18), applied to the output of the SSIA module in the next step, allowed to further improve all evaluation metrics, compared to the previous step. In the final two steps, with the inclusion of the DSFF modules (without and with the *local* loss applied to the SSIA output), the best values of all evaluation metrics were achieved at the latter step, except for *recall* on the Kvasir-SEG dataset. This indicates that, guided by the local feature graphs, the DSFF modules allow the network to effectively focus on target area features, reducing irrelevant area weights and contributing to the improvement in segmentation performance.

### 3.3 Performance comparison with state-of-the-art segmentation networks

Finally, we compared the segmentation performance of the proposed DLGRAFE-Net network with that of state-of-the-art networks, based on their results reported in the corresponding literature sources. The results are shown in Tables 6 and 7, respectively for the CVC-ClinicDB dataset and Kvasir-SEG dataset. As can be seen from Table 6, DLGRAFE-Net outperforms all state-of-the-art networks on the CVC-ClinicDB dataset according to the two most important evaluation metrics in the field of image segmentation, namely *DSC* and *IoU*. Based on *precision* and *recall*, DLGRAFE-Net takes second place here. On the Kvasir-SEG dataset, the superiority of the proposed network is even more evident as it outperforms all state-of-the-art networks according to three (out of four) evaluation metrics, including the two most important ones, i.e., *DSC* and *IoU*. Only based on *recall*, DLGRAFE-Net takes fourth place here.

**Table 6:** CVC-ClinicDB dataset’s segmentation results based on literature sources.

Network	*DSC* (%)	*Precision* (%)	*Recall* (%)	*IoU* (%)
DeepLabV3+ [41]	89.30	91.90	89.40	84.30
PolypSegNet [42]	91.52	**96.21**	91.13	86.22
BSCA-Net [43]	92.60	-	-	87.40
TranSEFusionNet [28]	86.48	-	-	79.09
ConvSegNet [26]	91.77	90.48	**95.18**	86.50
PraNet [24]	89.50	89.90	91.20	85.30
HarDNet-CPS [35]	91.70	-	-	88.70
ECTransNet [29]	92.30	93.30	93.10	87.80
DLGRAFE-Net (*proposed*)	**94.38**	94.24	95.10	**89.96**

**Table 7:** Kvasir-SEG dataset’s segmentation results based on literature sources.

Network	*DSC* (%)	*Precision* (%)	*Recall* (%)	*IoU* (%)
DeepLabV3+ [41]	89.00	92.00	88.50	83.10
PolypSegNet [42]	88.72	91.68	**92.54**	82.56
BSCA-Net [43]	91.00	-	-	85.50
TranSEFusionNet [28]	84.51	-	-	78.10
ConvSegNet [26]	86.18	86.92	91.24	79.36
PraNet [24]	89.60	92.10	89.40	83.80
HarDNet-CPS [35]	91.10	-	-	85.60
ECTransNet [29]	90.10	93.20	89.00	83.90
DLGRAFE-Net (*proposed*)	**91.66**	**95.44**	89.35	**85.64**

## 4. Conclusion

A Double Loss Guided Residual Attention and Feature Enhancement Network (DLGRAFE-Net) has been proposed in this paper for polyp segmentation. Through an effective combination of residual networks and feature fusion modules, DLGRAFE-Net significantly enhances the feature fitting of neural networks, capturing the positional and shape edge features of polyps, thereby further improving segmentation performance as evident from the provided experimental results obtained on two public datasets.

Despite the success of DLGRAFE-Net in polyp segmentation, there are still several unresolved issues. For instance, we need to elaborate more effective preprocessing methods, adopting targeted approaches such as removing artifacts and noise, and performing image registration, which would contribute to enhancing the segmentation performance. Although the network proposed in this paper has significant advantages in terms of accuracy, reducing the computational complexity is also a difficult point for us to break through in the future. Addressing distribution differences between datasets is also a challenging problem worth further investigation. By tackling these issues, we can achieve more reliable polyp segmentation, which will have a positive impact on the field of medical image segmentation and clinical applications.
